# Angiotensin Converting Enzyme (ACE) and ACE2 Bind Integrins and ACE2 Regulates Integrin Signalling

**DOI:** 10.1371/journal.pone.0034747

**Published:** 2012-04-16

**Authors:** Nicola E. Clarke, Martin J. Fisher, Karen E. Porter, Daniel W. Lambert, Anthony J. Turner

**Affiliations:** 1 Institute of Molecular and Cellular Biology, Faculty of Biological Sciences, University of Leeds, Leeds, United Kingdom; 2 Department of Chemistry, University of Leeds, Leeds, United Kingdom; 3 Multidisciplinary Cardiovascular Research Centre, University of Leeds, Leeds, United Kingdom; 4 Oral Disease Research Group, University of Sheffield, Sheffield, United Kingdom; Lerner Research Institute, United States of America

## Abstract

The angiotensin converting enzymes (ACEs) are the key catalytic components of the renin-angiotensin system, mediating precise regulation of blood pressure by counterbalancing the effects of each other. Inhibition of ACE has been shown to improve pathology in cardiovascular disease, whilst ACE2 is cardioprotective in the failing heart. However, the mechanisms by which ACE2 mediates its cardioprotective functions have yet to be fully elucidated. Here we demonstrate that both ACE and ACE2 bind integrin subunits, in an RGD-independent manner, and that they can act as cell adhesion substrates. We show that cellular expression of ACE2 enhanced cell adhesion. Furthermore, we present evidence that soluble ACE2 (sACE2) is capable of suppressing integrin signalling mediated by FAK. In addition, sACE2 increases the expression of Akt, thereby lowering the proportion of the signalling molecule phosphorylated Akt. These results suggest that ACE2 plays a role in cell-cell interactions, possibly acting to fine-tune integrin signalling. Hence the expression and cleavage of ACE2 at the plasma membrane may influence cell-extracellular matrix interactions and the signalling that mediates cell survival and proliferation. As such, ectodomain shedding of ACE2 may play a role in the process of pathological cardiac remodelling.

## Introduction

Heart failure is characterised as a decline in cardiac contractility, which is associated with structural changes, collectively termed cardiac remodelling. Cardiac myofibroblasts are key mediators of cardiac remodelling via their proliferation, invasion and secretion of extracellular matrix proteins. Angiotensin II (Ang II) stimulates cardiac myofibroblast transdifferentiation leading to fibrosis. Ang II also stimulates proliferation [1], NADPH oxidase activation [2] (and thereby reactive oxygen species production), the production of proinflammatory cytokines [3] and the activation of matrix metalloproteinases (MMPs) [4]. As a result, Ang II is a major contributor to the pathology of cardiovascular diseases. Ang II is generated from the biologically inert peptide, Ang I, by the catalytic action of angiotensin converting enzyme (ACE), a key proteolytic step in the renin angiotensin system (RAS). Aberrant functioning of the RAS is a feature of a variety of cardiovascular, renal and other pathologies and ACE inhibitors and Ang II receptor 1 (AT1R) antagonists are widely used in the clinic. Accordingly, ACE inhibition has been shown to prevent cardiac remodelling after myocardial infarction (MI) and preserves cardiac function [5], [6]. A combination of ACE inhibitors and AT1R blockers has been shown to be more effective than either alone [7]. A decade ago a new member of this system was identified, termed angiotensin converting enzyme 2 (ACE2) [8], [9]. ACE2 acts to hydrolyse Ang II into the vasodilator Ang-(1-7), thereby contributing to reductions in blood pressure. Current models of the RAS are based on the concept that the two enzymes counterbalance each other.

The balance between the two angiotensin converting enzymes has been highlighted by ACE2 deletion murine models, which have a significantly higher mortality rate post-MI than wild-type mice. Mortality was associated with enhanced adverse ventricular remodelling following MI [10], a state which was reversed by the use of an AT1R blocker and as such the pathology of ACE2 deletion was attributed to the increased levels of Ang II [10]. A mounting body of evidence is forming in support of a cardioprotective role for ACE2, through the metabolism of Ang II [10], [11], but also through the direct action of Ang-(1-7) via its own receptor, Mas [12]. Like Ang II the actions of Ang-(1-7) extend beyond vasopressor control, and for the most part appear to counteract the effects of Ang II and therefore mediate cardioprotection [13]. Ang-(1-7) reduces interstitial fibrosis [14], myocyte hypertrophy [15] and inhibits myocyte cell growth [16]. The reduction in myocyte hypertrophy resulting from expression of Ang-(1-7) was associated with a decrease in pro-inflammatory cytokines (TNF-α and IL-6) and also a reduction in exogenous ACE transcript [17].

Both ACE and ACE2 are increased in the failing heart [18]–[20]. Over-expression of ACE2 and inhibition of ACE exert a protective influence on the heart post-myocardial infarction (MI) and prevent the pathological remodelling [21]. These data together suggest that the regulated activity of angiotensin converting enzymes may play a role in cardiac homeostasis.

In addition to its catalytic actions, ACE2 is the cellular receptor for the SARS virus; more recently, other regulatory actions of ACE2 through protein-protein interactions have been identified [22]. ACE2 acts as a chaperone protein for the neutral amino acid transporter, B^0^AT, [23] and binding of calmodulin to the cytoplasmic tail of ACE2 regulates its retention on the cell surface [24]. The reported observation that ACE2 binds integrin β1 (ITGB1) in the failing human heart [25] adds another dimension to the role of the ACE family in cardiac homeostasis.

Integrins are a family of αβ heterodimeric cell surface receptors, which link extracellular matrix proteins with the intracellular cytoskeleton. Integrins have an important role in the regulation of gene expression, cell proliferation, differentiation, migration and apoptosis. Activated myofibroblasts develop specialised focal adhesions, containing high levels of α5, β1 and β3 integrins [26]. ITGB1 serves as a mechanotransducer and expression of this integrin increases in the heart after MI [27]. ITGB1 is highly implicated in left ventricular remodelling and MI models have shown it is essential for the adaptive remodelling response [28] leading to the suggestion that the functional activity of ACE2 in cardiac remodelling may, at least in part, be mediated through ITGB1.

Both the angiotensin converting enzymes and ITGB1 traverse the plasma membrane; the angiotensin converting enzymes also exist in soluble forms in the plasma when shed from the cell membrane. Recent studies of heart failure patients have linked elevated levels of soluble (shed) ACE2 (sACE2) to increased myocardial dysfunction and thus have indirectly identified a protective role for the cell surface-associated form [29]. Hence, the ectodomain of ACE2 may have a role in cardiac remodelling independent of its catalytic activity. Here we show that both angiotensin converting enzymes bind ITGB1 and integrin α5 (ITGA5) *in vitro*. The data presented provide evidence that the enzymes provide a substrate for cellular adhesion and that the interaction between ACE2 and an integrin increases cellular adhesion when both are present on the cell membrane. We demonstrate that the ectodomain of ACE2 regulates integrin induced cell signalling via modulation of the phosphorylation of focal adhesion kinase (FAK) and Akt expression levels.

## Results

### ACE2 binds Integrin β1

**Figure 1:**
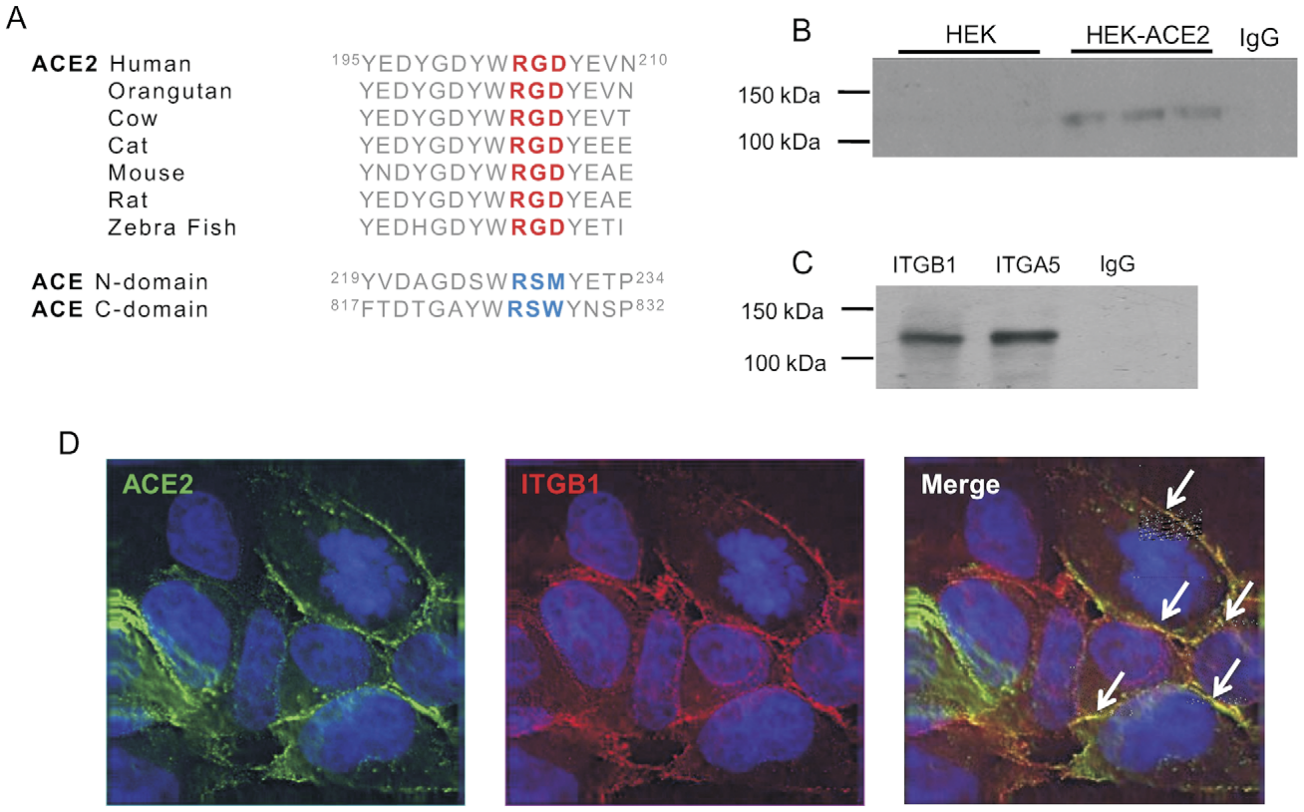
ACE2 binds an integrin. *A)* Evolutionary alignment of a part of the ACE2 amino acid sequence. ACE2 protein sequence conservation surrounding and including the proposed integrin binding site. Amino acids are shown as their one letter codes. The predicted integrin binding site is highlighted in red, whilst the homologous region in ACE is coloured blue. This alignment of the ACE2 protein sequence was taken from the Uniprot database. *B)* Immunoprecipitation of ITGB1 with ACE2 in HEK-ACE2. HEK cell lysates incubated with ITGB1 antibody and eluted using protein G. Immunoblotting for ACE2 was performed with anti-ACE2 antibody. *C)* Immunoprecipitation of ITGB1 and ITGA5 with ACE2 in Huh7 cell monolayers. Cell monolayers were cross-linked using DTBP before lysis and immunoprecipitation as before. *D)* Immunocytochemical detection of ACE2 and ITGB1 location in HEK-ACE2 cells. Cell imaging shows ACE2 (green) and ITGB1 (red) are located together (yellow) on the cell membrane of HEK-ACE2 cells.

A typical integrin binding motif is the tripeptide sequence RGD. Bioinformatic analysis was used to compare the protein sequences of ACE and ACE2; a highly conserved integrin binding domain was identified in the ectodomain of ACE2 but not ACE (Figure 1A). There are two isoforms of ACE: somatic ACE, that contains two homologous catalytic ectodomains (the N and C-domains) and the testicular ACE (tACE), composed solely of the C-domain. The RGD sequence in ACE2 is replaced by the sequence RSW in the ACE N-domain and RSM in the C-domain.

To assess the ability of ACE2 to bind an integrin, immunoprecipitation was performed on HEK cells over-expressing ACE2 (HEK-ACE2 cells). Anti-ITGB1 antibody was used to pull down any interacting proteins and western blotting performed using an anti-ACE2 antibody (Figure 1B). An interaction was found to occur in the HEK-ACE2 cells, represented by a single band at 120 kDa corresponding to the fully glycosylated ACE2 protein (Figure 1B). This interaction was specific, since ACE2 was not detected when immunoprecipitation was performed with isotype control IgG antibody.

Immunoprecipitation was repeated in Huh7 cells, which endogenously express ACE2. Crosslinking was performed in order to fix any interaction of less than 9 Å in length. An interaction between ACE2 and ITGB1 was readily detected in these cells (Figure 1C). In addition an interaction between ACE2 and the common binding partner of ITGB1 in cardiac tissue, ITGA5, was probed by subjecting cell lysates to immunoprecipitation with anti-integrin α5 (ITGA5) antibody. ITGA5 was also found to bind to ACE2 in Huh7 cells (Figure 1C). The subcellular location of both ACE2 and ITGB1 was visualised in HEK-ACE2 cells by immunofluorescence microscopy (Figure 1D). Antibodies to ACE2 (green) and ITGB1 (red) located both proteins to the plasma membrane. Co-location of the two proteins was observed in some areas (yellow), highlighted by arrows.

### Both ACE and ACE2 bind integrins independently of an RGD sequence

**Figure 2:**
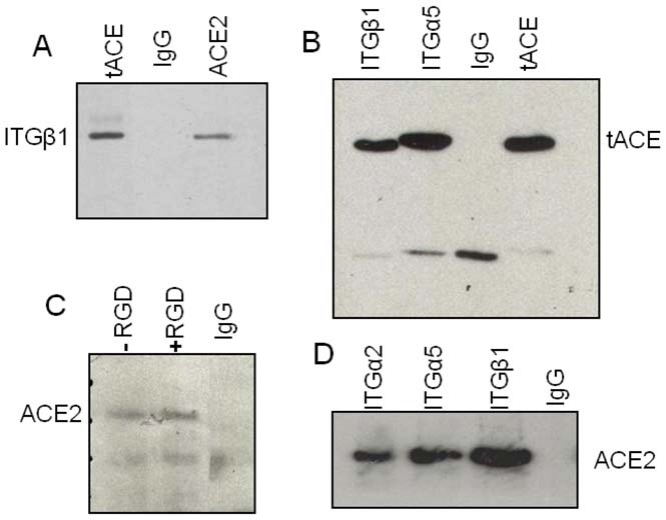
Both angiotensin converting enzymes bind integrins independent of an RGD motif. *A)* Immunoprecipitation of ITGB1 with tACE in HEK-tACE cells. HEK cell lysates were incubated with ITGB1 antibody and eluted using protein G. Immunoblotting for ACE was performed with anti-ACE mC5 antibody. This interaction was similar to that between ACE2 and ITGB1 in ACE2 over-expressing cells. *B)* Immunoprecipitation of ITGB1 and ITGA5 with tACE in SHSY5Y cells over-expressing the testicular form of ACE. Immunoprecipitation was performed as before. *C)* Immunoprecipitation of ACE2 with ITGB1 in the presence of a RGD peptide. Huh7 cells were pre-incubated and cross-linked using DTBP, in the presence or absence of RGD peptide before lysis and immunoprecipitation as before. *D)* Immunoprecipitation of ITGA2, ITGA5, ITGB1 with ACE2 in Huh7 cell monolayers. Cell monolayers were cross-linked using DTBP before lysis and immunoprecipitation as before.

Both isoforms of ACE lack the RGD motif present in the extracellular domain of ACE2 and we therefore hypothesised that ACE would not bind integrins. Cells over-expressing tACE were used to examine any potential interaction between ACE and ITGB1. Immunoprecipitation revealed that ACE does bind ITGB1 (Figure 2A) and that ACE also binds ITGA5 similarly (Figure 2B). In order to clarify any role of the RGD motif in the binding of ACE2 to integrins, cross-linked immunoprecipitation was repeated in Huh7 cells, in the presence and absence of an RGD peptide. The interaction between ACE2 and ITGB1 was not blocked by the presence of RGD peptide (Figure 2C). Hence, the ability of ACE2 to bind the RGD-independent integrin subunit ITGA2 was additionally investigated. ACE2 bound ITGA2 at a comparable level to ITGA5 and ITGB1 (Figure 2D).

### The RGD motif is inaccessible in ACE2

**Figure 3:**
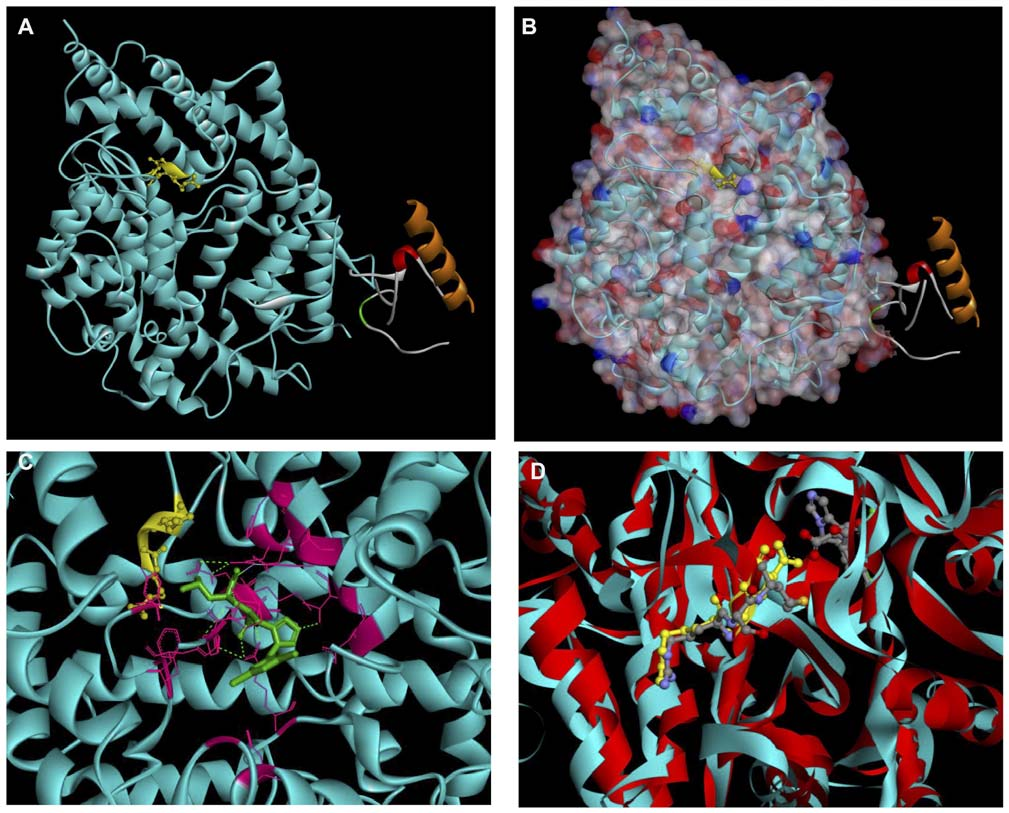
The RGD motif in ACE2 is partially inaccessible as the aspartate residue is buried in the active site cleft. *A)* ACE2 Ribbon structure RGD sequence highlighted in yellow. *B)* Space filling model showing arginine and glycine of the RGD motif are accessible on the protein surface, whilst the aspartate protrudes into the active site cleft. *C)* Close up image of the α-carbon trace of the inhibitor bound ACE2 structure looking into the active site (obscuring unstructured loops removed). The coordinating active site residues stabilising the inhibitor, Mln-4760. *D)* Alignment of the inhibitor bound structures of ACE and ACE2. α-carbon trace of the inhibitor bound structures looking into the active site cleft. The RGD motif in ACE2 and the RSM motif in tACE are shown as ball and stick representation and the inhibitor, Captopril, illustrating the location of the substrates in the active site of both proteins. Structure taken from PDB.org, file 1r42; manipulated with Accelrys DS visualiser 2.0. Turquoise, ACE2 extracellular domain (residues 1–615); red, tACE; yellow, RGD motif; green, Mln-4760; lilac, zinc; pink, active site residues; all other colours in the ribbon structure are sections of the collectrin homology domain disordered in solution. In the spacing filling model: Overall surface, light pink; hydrogen bond acceptors, red; hydrogen bond donors, blue.

We utilised molecular modelling to ascertain the location of the RGD motif in the structure of ACE2 to investigate the apparent functional redundancy of this motif in integrin binding. Examination of the ACE2 structure *in silico* [30] revealed that the motif was on the protein surface (Figure 3A). However, the space filling model revealed that the aspartate residue of the RGD motif faces into the active site cleft (Figure 3B and 3C) and is, therefore, inaccessible for protein-protein interactions. The RSM sequence in tACE superimposes in exactly the same position as the RGD sequence in ACE2 and is therefore also inaccessible for protein-protein interactions (Figure 3D). The similarity between the two proteins is illustrated by the close proximity of the outline trace and the degree of overlap. Variations in amino acid sequences are illustrated by the slight offset in the α-helical loops.

### ACE2 can act as a cell adhesion substrate

To examine the functional significance of an interaction between the angiotensin converting enzymes and integrins, adhesion assays were used to explore the possibility that they may act as a cell adhesion substrate. We designed an *in vitro* technique representative of an *in vivo* cellular environment to study the effect of ACE2 expression on cellular attachment.

**Figure 4:**
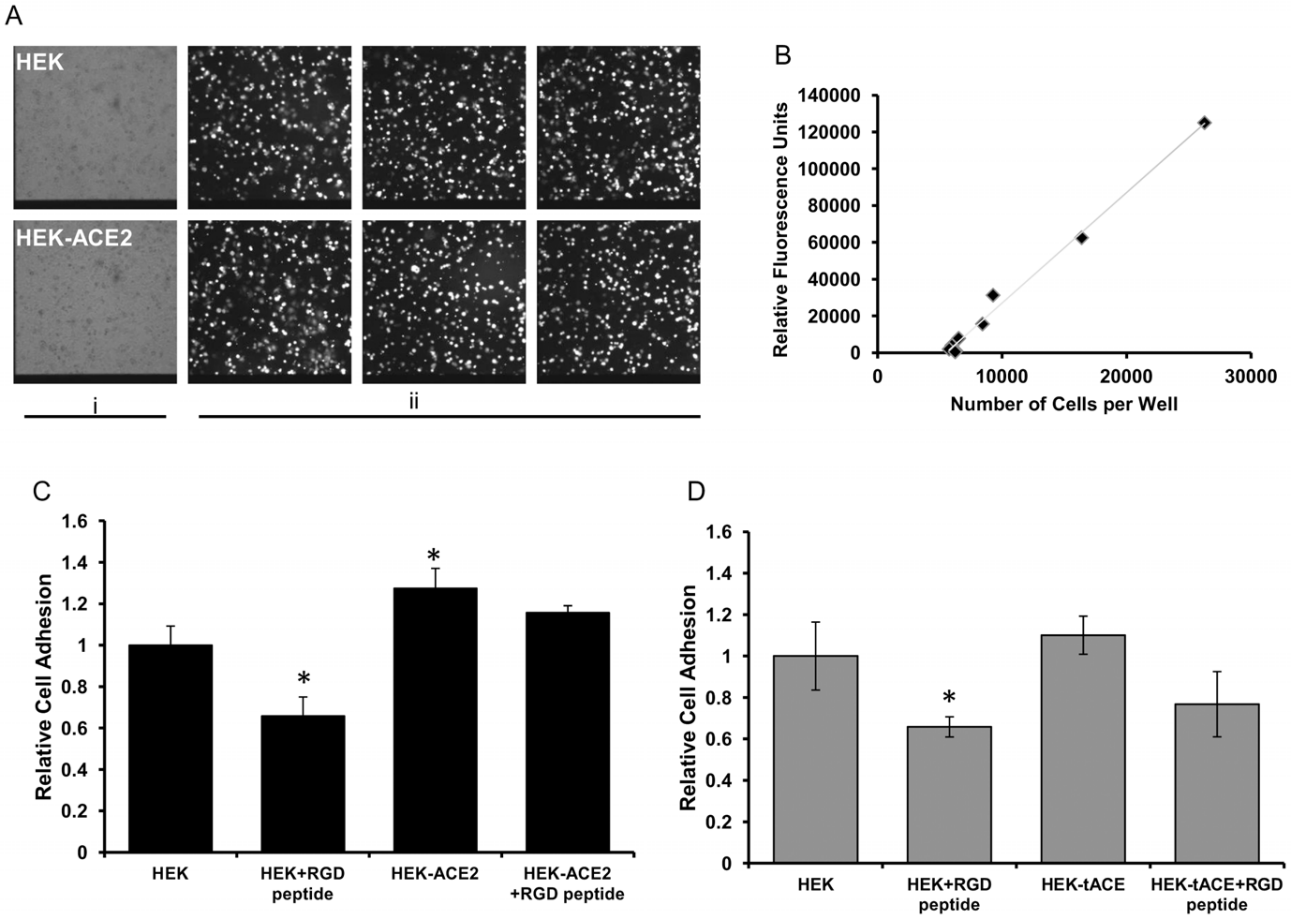
ACE2 affects cellular adhesion *in vitro*. Huh7 cells were loaded with BCECF and plated on to monolayers of HEK, HEK-ACE2 or HEK-tACE cells, unadhered cells were washed off. *A)* Cell adhesion was examined using fluorescence microscopy, Panel i, HEK and HEK-ACE2 substrates were of equal confluence. Panel ii, labelled cells were visualised at 450–480 nm excitation wavelength. *B)* Fluorescent intensity increases linearly with the number of cells plated. Serial dilutions were made of the labelled Huh7 cells and fluorescence was read. *C)* Cellular expression of ACE2 increases cell adhesion independent of RGD motif. Cells were treated as before or the HEK-ACE2 cells were pre-incubated with RGD peptide before Huh7 cell addition. The number of cells which had adhered was quantified by fluorescent emission. *D)* Cellular expression of tACE does not affect cell adhesion. Cells were treated as before, the number of cells which had adhered was quantified by fluorescent emission.

A cell to cell adhesion assay was developed in order to examine the ability of membrane bound ACE or ACE2 to act as a ligand for cell adhesion. Cells over-expressing ACE or ACE2 or their mock transfected controls were used as cell adhesion substrates and Huh7 cells were labelled with BCECF fluorescent dye and allowed to adhere. Huh7 cell adhesion to the substrate cells was confirmed by immunofluorescence microscopy (Figure 4A). Calibrations confirmed that the relative fluorescence measured was proportional to the number of cells seeded (Figure 4B). A significant difference was seen between the adhesion of Huh7 cells to ACE2-expressing cells compared with non ACE2-expressing cells (Figure 4C); the expression of ACE2 on the cell surface increased cell adhesion by approximately 25%. To further examine any role of RGD-mediated cell adhesion, cells were incubated with an RGD peptide prior to plating. Pre-incubation with RGD peptide significantly reduced the adhesion of Huh7 to HEK control cells (Figure 4C); cellular expression of ACE2 abolished the decrease mediated by pre-incubation with an RGD peptide (Figure 4C). Conversely, cellular expression of ACE conferred no enhanced adhesion properties compared to control cells (Figure 4D). In this model the presence of an RGD peptide reduced cellular adhesion by approximately 35% independent of tACE expression (Figure 4D).

**Figure 5:**
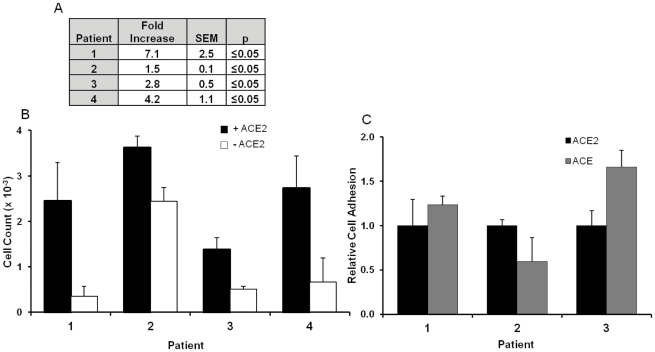
The angiotensin converting enzymes are cell adhesion substrates. Microtitre plates were coated with or without sACE2 and blocked with BSA (1%). Wells were washed; CmF cells plated and allowed to adhere (2.5 h at 37°C). After incubation unadhered cells were washed off and cell adhesion was quantified using MTS reagent and reference to a calibration curve. *A)* Tabulated cell adhesion results for different patients. *B)* Graphical representation of *(A)*. *C)* Cell adhesion is comparable between ACE and ACE2. Plates were coated with sACE2, ACE or PBS and adhesion assays performed as before.

To examine the physiological importance of the adhesion properties of ACE2 and ACE, adhesion assays were performed using primary human cardiac myofibroblast (CF) cells, important mediators of cardiac remodelling. All cells adhered strongly to fibronectin (data not shown), an important component of the extracellular matrix, adhesion to which is integrin-mediated [31]. A significant difference in cell binding was seen in the presence and absence of ACE2 (Figure 5A) in patient samples. The average fold increase in cell adhesion in the presence of ACE2 was 3.9 fold and *p* = 0.0035. Differences in adhesion to both fibronectin and ACE2 were seen in all patient cells (Figure 5A); this is likely due to an inherent variation in integrin and/or ACE2 expression levels since these were primary cells [32]. As previous experiments had shown ACE2 and ACE both bind integrins, investigations were performed to determine if ACE could also act as a cell substrate. Cells adhered comparably to both ACE and ACE2 (Figure 5B) and the presence of an RGD peptide had no effect on the adhesion to ACE or ACE2 (data not shown).

### Signalling properties of ACE2

**Figure 6:**
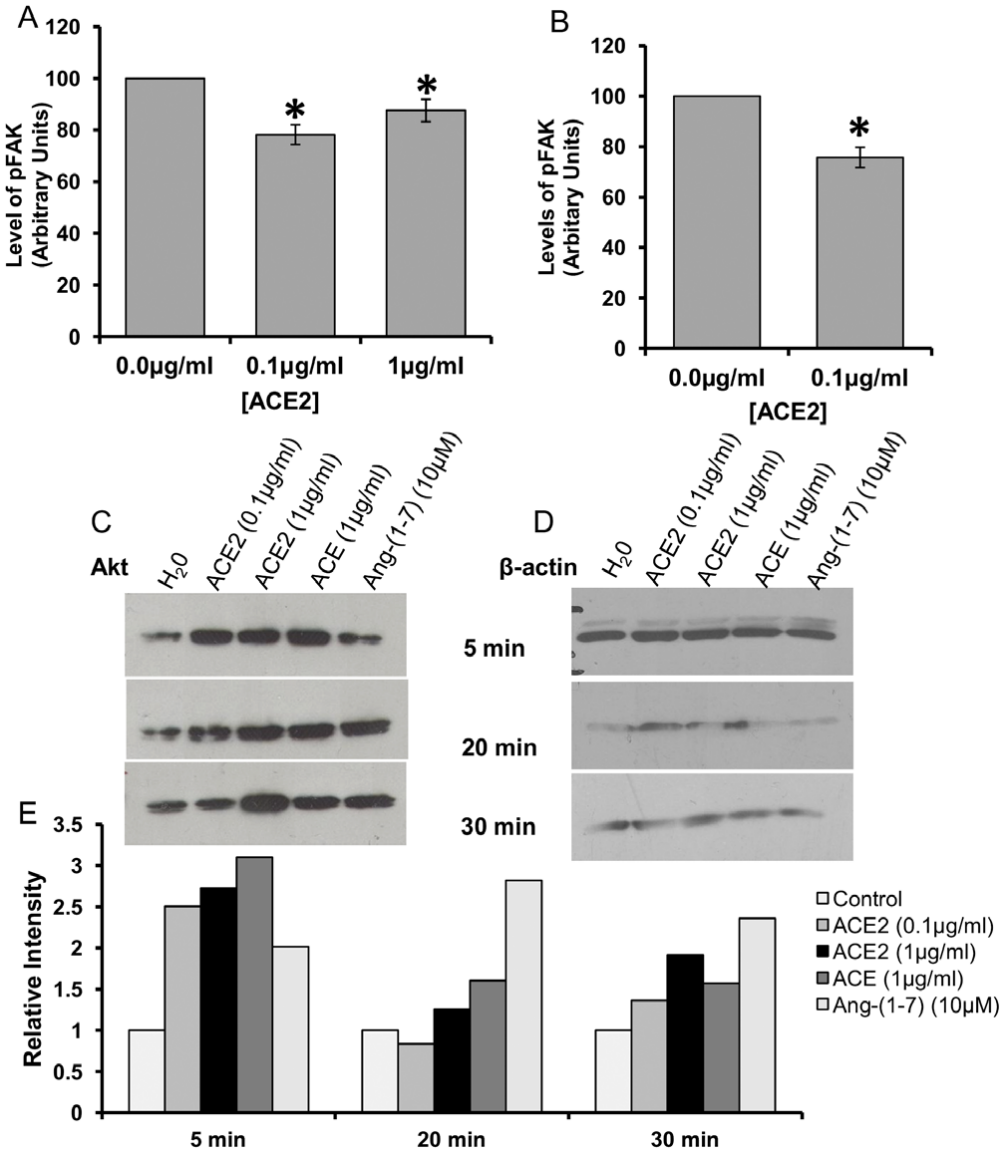
sACE2 inhibits integrin signalling. *A &B)* Treatment with sACE2 decreases the level of pFAK. Huh7 cells *(A)* or CF cells *(B)* were starved for 12 hours, then treated in the presence or absence of sACE2 at concentration indicated for 20 min. Significance determined by one-way ANOVA (Huh7) or Students' t-test (CF) where *p≤0.05. Following treatment with sACE the level of pFAK within the cells was assayed using an ELISA kit as per manufacturer's instructions. *C, D&E)* Treatment with sACE2 increases cellular Akt expression. Cells were treated as above with either: sACE2, ACE, or Ang-(1-7), at the concentration and for the length of time indicated. Levels of Akt *(C)* and β-actin *(B)* were visualised by immunoblot and quantified by densitometric analysis *(E)*.

In light of the association of ACE2 with integrins, experiments were performed to determine if ACE2 could elicit integrin signalling. Focal adhesion kinase (FAK) is stimulated early in any integrin signalling cascade. Given that the extracellular domain of ACE2 binds integrin, cells were stimulated with the ectodomain of ACE2. The levels of phosphorylated FAK (pFAK) in the presence and absence of sACE2 were quantified by ELISA. At 0.1 µg/ml sACE2 significantly reduced levels of pFAK in Huh7 cells and in primary myofibroblasts (Figure 6A and B). No further decrease was observed when cells were incubated with 1 µg/ml ACE2. Downstream translation of this signal to Akt was investigated by quantifying the levels of phosphorylated Akt. Western blot analysis revealed that levels of phosphorylated Akt in Huh7 cells increased in response to stimulation with sACE2 and Ang II for 30 min (data not shown). However, this increase was accounted for by an up-regulation in Akt protein expression (Figure 6). The catalytic product of ACE2, Ang-(1-7), alone did not elicit the same effects as ACE2 (Figure 6).

**Figure 7:**
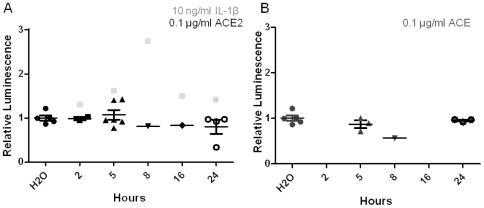
sACE2 does not affect NF-κB levels. *A)* Huh7 cells were co-transfected with NF-êB luciferase reporter and TK-Renilla luciferase (control) plasmids. 24 h post-transfection, cells were stimulated with, sACE2/ACE, 100 ng/ml, or IL-1β, 100 ng/ml diluted in DMEM containing 1% FCS. After treatment, Luciferase levels were analysed using Dual-Luciferase Reporter Assay System following manufacturer's instructions.

Further analysis revealed that this signal was not transmitted to the downstream effector of Akt signalling, NF-κB. Cells were transfected with NF-κB reporter vector. Treatment of cells with sACE2 or ACE for between 2 and 24 h resulted in no significant change in luminescence compared to control (Figure 7). Furthermore, neither Ang II nor Ang-(1-7) had any significant effect (data not shown).

## Discussion

The present study reveals that both ACE and ACE2 bind integrins in an RGD-independent manner. We have shown that ACE2, in particular, increases cellular adhesion and, moreover, affects integrin signalling. Shedding of the ACE2 protein may relieve repression of integrin signalling, exerted by the presence of ACE2 on the cell membrane. Using primary cardiac myofibroblasts we have demonstrated that the actions of ACE and ACE2 exerted on cell models are physiologically relevant to the diseased human heart.

Both ACE and ACE2 are increased in the failing heart [18]–[20]. ACE2 expression has consistently been seen to be up-regulated in the peri-infarct area after MI [10], [20], [33] and in end stage heart failure [34] indicative of a role in injury. Knockout of the *ace2* gene increases MMP2 and MMP9 levels in the peri-infarct region of mice, resulting in disruption of the extracellular matrix and enhanced adverse remodelling [10]. ACE2 over-expression has been shown to inhibit collagen production in response to hypoxic injury [35]. Moreover, activation of remodelling pathways has been demonstrated in *ace2* knockout animals in the absence of an increase in Ang II [36]. Cardiac remodelling is a key pathological process in the development of heart failure. Integrins play a key role in this process by mediating cell-ECM interactions and cellular signalling.

ITGB1 has also been implicated in myocardial dysfunction [28]. An association between ITGB1 and ACE2 has previously been reported in the failing heart and attributed to the presence of an RGD motif in ACE2 [25]. We have established that both ACE and ACE2 binds ITGB1 and also its common cardiac binding partner, ITGA5 [37], as well as the RGD-independent and liver rich ITGA2 [38]. However, in contrast to the study of Lin et al. [25] our data clearly suggest that these interactions occur independently of an RGD motif. 3-dimensional modelling has demonstrated that the RGD motif present in the ectodomain of ACE2 is inaccessible, which explains its redundancy in integrin binding. The aspartate residue is positioned facing the active site cleft of the protein and as such is not available to bind into the integrin binding pocket [39]. Given the structural homology between the two proteins, it is not surprising that ACE is also capable of binding integrins as the interaction appears to be independent of the RGD sequence, which is lacking in ACE.

Prothrombin, like ACE2, contains a partially buried RGD motif, however in prothrombin this sequence is exposed upon activation. The ability of prothrombin to bind integrin αvβ3 is key to its biological activity in the fibrotic cascade [40]. As in ACE2, the carboxylate group of the Asp of the RGD motif is directed towards the specificity pocket of the enzyme [41]. In its native state prothrombin does not exert strong adhesive properties. However, proteolytic maturation exposes the RGD motif and thereby enhances the adhesive properties of thrombin [42]. This mechanism is unlikely to occur in ACE2 which, when present on the cell membrane, is in its mature form (unlike thrombin it does not occur in an inactive proenzyme form during biosynthesis). What is more, any rearrangement of the active site of ACE2 is likely to inhibit its catalytic activity; sACE2 is catalytically active.

Although RGD motifs are the most common mechanism of integrin binding, there are other cell surface proteins which bind integrins despite lacking an RGD motif. ADAM 9, for example, binds through a hypervariable loop stabilised by disulphide bridges, which protrudes from the surface of the protein structure [43], [44]. The cellular adhesion molecule ICAM-1 is hypothesised to bind via immunoglobulin type domains [45], whereas other proteins have been predicted to bind through a (D/E)ECD motif [46]. Neither ACE nor ACE2 contain an ECD motif.

We have shown that the integrin binding to ACE2, but not ACE, is essential for the role of ACE2 as a cellular anchor when expressed on the cell surface. What is more, the presence of ACE2 on the cell surface partially removed the requirement of RGD-interactions for cellular adhesion. Fibroblast motility is a key process in the development of scar tissue and thus cellular adhesion is an important homeostatic mechanism. In order to study the role of ACE and ACE2 in the remodelling response, primary cardiac myofibroblasts were used as a disease model. These cells play a key role in the maintenance of cardiac architecture under conditions of injury, by forming scar tissue through their ability to proliferate and adhere [47]. We demonstrate that both ACE and ACE2 exert comparable effects over myofibroblast adhesion, but that again this effect is not mediated through an RGD motif. Recent clinical investigations have highlighted that elevated levels of sACE2 in patient plasma correlated with increased myocardial dysfunction [29] and vascular compliactions in type 1 diabetes [48]. We have shown that both ACE and ACE2 interact with the cell surface in adhesion assays and, furthermore, ACE2, in particular, enhances cell adhesion and may modulate integrin signalling. Cellular retention of ACE2 is therefore required for its role as a cellular anchor and, as such, the cleavage of ACE2 by ADAM17 [49] may be a pathological step in the development of heart failure. The retention of ACE2 on the cell membrane is known to be regulated by cell signalling [24] and viral infection [50]. Shed ACE2 could activate integrins by binding to them and transducing activating signals or, additionally, by interacting with non-integrin sites given the multiple protein-protein interactions with which the ACE2 protein is involved.

FAK is a critical signalling component associated with areas of substratum adhesion; signalling via FAK is mediated through autophosphorylation of Tyr^397^ [51]. We demonstrate that sACE2, at levels comparable to those reported in human plasma, significantly reduces FAK phosphorylation levels. Furthermore, we show that treatment with sACE2 increases the levels of Akt expression, a pro-survival, pro-proliferative protein. Changes in the level of phosphorylated Akt were also seen in response to sACE2; however, these were accounted for by the increase in the amount of total Akt. As such, signalling by Akt was not transmitted to its downstream effector NF-κB. sACE2 has no known function. We do not dismiss the possibility that this circulating ACE2 may bind integrins, when released from the plasma membrane, and elicit autocrine or paracrine signalling. In fact, one of the shed forms of the amyloid precursor protein, sAPPα, binds to ITGB1 and signals to enhance axon outgrowth [52]. What is more, cellular expression of full length APP inhibits axonal outgrowth and an excess of the shed form overcomes this inhibition [52]. We therefore propose some of the anti-proliferative actions of ACE2 may in part be mediated through a non-catalytic interaction with integrins, rather than by metabolism of Ang II *per se*. These data suggest that ACE2, through its integrin binding abilities, may have regulatory roles in cellular attachment and support a novel mechanism of integrin activation upon ACE2 shedding.

## Materials and Methods

### Materials

All routinely used reagents were purchased from Sigma unless otherwise stated. Cell culture reagents were purchased from Lonza (Slough, UK). Cell Extraction Buffer and Elisa (pFAK) kit were purchased from Invitrogen (Paisley, UK) along with lipofectamine and BCECF reagent (2′7′-bis-(2-carboxyethyl)-5-(and6)-carboxyfluorescein). Roche (Welwyn, UK) supplied protease inhibitor tablets. Fluorescent ACE2 substrate Mca-APK-Dpn was supplied by Enzo (Exeter, UK) and MTS reagent by Promega (Southampton, UK). Secondary antibodies, the chemiluminescence system used and Protein G Sepharose 4 fast flow were supplied by GE healthcare (Chalfont St. Giles, UK). DTBP (Dimethyl 3,3′-dithiobispropionimidate) was purchased from Pierce (Cramlington, UK). The ACE2 inhibitor 416F2 [53] was a generous gift from Prof V. Dive (CEA, Gif sur Yvette, France). Polyclonal ACE2 antibody raised in goat was purchased from R&D systems (Abingdon, UK). Integrin antibodies raised in mice against ITGB1 and ITGA5 antibodies were bought from Santa Cruz Biotech (Heidelberg, Germany). Polyclonal ADAM17 antibody raised in rabbit was purchased from Calbiochem (Nottingham, UK), while Akt antibody raised in rabbit was purchased from Cell Signaling (Hertfordshire, UK). Purified ACE enzyme was a kind gift from Prof N. Hooper (The University of Leeds, UK) and Prof S. Danilov (University of Illinois at Chicago, USA) generously provided the ACE monoclonal antibody [54], [55].

### Cell culture and isolations

HEK (human embryonic kidney) and Huh7 (hepatocellular carcinoma-derived) [24] cells were cultured in Dulbecco's modified Eagle's medium (DMEM), supplemented with 10% (v/v) foetal bovine serum, 2 mM essential amino acids, 1% (v/v) non-essential amino acids. HEK cells stably transfected with full length ACE2, designated HEK-ACE2 [49], and those overexpressing the testicular form of ACE (tACE), HEK-tACE [56], were cultured in the same conditions with the addition of G418 (0.5 mg/ml) to the medium. SHSY5Y cells were cultured in DMEM-F12 media [57], and SHSY5Y cells overexpressing tACE, SHSY5Y-tACE cells, were a kind gift from Dr. C. Rushworth, and were cultured in DMEM-F12 supplemented with G418 (0.5 mg/ml).

Cardiac myofibroblasts were obtained by enzymatic digestion of biopsies of human right atrial appendage. Patients were undergoing elective coronary artery bypass surgery and had normal ventricular function (ejection fraction normal (≥50% by cardiac ultrasound and/or LV Angiography). Local (Leeds West) Research Ethical Committee (LREC) approval is in place for this study; written consent was given, reference number 01/040. Informed, written patient consent is obtained. The investigation conformed to the principles outlined in the Declaration of Helsinki, 1997. Primary cultures of cardiac fibroblasts were harvested, characterized as myofibroblasts co-expression of smooth muscle α-actin and vimentin and cultured as described previously [58]. Experiments were performed on cells from different patients at passages 2–5 [59]. Cell images were taken on a Nikon Eclipse TS100 microscope using a Nikon COOLPIX 4500 4.0 megapixel camera.

### Recombinant ACE2 purification

HEK cells stably expressing a FLAG-tagged ACE2 ectodomain (sACE2) were created. HEK cells were transfected with plasmid DNA (pCl-neo containing nucleotides 104–2323 of ACE2 cDNA with the FLAG peptide conjugated to the C-terminus). Successfully transfected cells were selected by passage in media containing G418 (1 mg/ml). sACE2 was collected from the conditioned media of these cells and purified by affinity chromatography using an anti-FLAG M2-agarose column and eluted into tubes containing 25 µl 1M Tris pH 8.0 by addition of 0.1 M glycine, pH 3.5. Eluted fractions were analysed for ACE2 activity by fluorometric assay [24] and purity was checked by silver stain, using SilverXpress (Invitrogen) as per manufacturer's instructions, and then immunoblotted for ACE2.

### Cell treatments, transfections and lysis

Cells were treated at 80% confluency and all pharmacological reagents were diluted in OptiMEM. For pFAK quantifications and phospho-Akt quantifications, incubations with sACE2 were carried out with 100 ng/ml or 1 µg/ml sACE2 for the time indicated (pFAK, 20 min). After treatment, cells were placed on ice and lysed in either ice cold RIPA buffer containing protease inhibitor and phosphoSTOP (phosphor-Akt), or Cell Extraction Buffer (pFAK). Lysates were then analysed by pFAK ELISA as per manufacturer's instructions, or by western blot for Akt levels.

Huh7 cells were transfected with pGL4.32 [luc2P/NF-κB-RE/Hygro] (NF-κB reporter) vector (500 ng) using Lipofectamine 2000 in OptiMEM. Renilla CMV (1 ng) was co-transfected. Cell medium was changed 4 h post-transfection and stimulated after 24 h with either sACE2/ACE, 100 ng/ml, IL-1β, 100 ng/ml, diluted in DMEM containing 1% FCS. After treatment, luciferase levels were analysed using Dual-Luciferase Reporter Assay System following manufacturer's instructions.

Lysates were routinely prepared by solubilisation of cells in RIPA buffer (0.1 M Tris–HCl, pH 7.4, 0.15 M NaCl, 1% (v/v) Triton X-100, 0.1% (v/v) Nonidet P-40). Protein concentrations were determined by bicinchoninic acid (BCA) protein assay [60]. Bovine serum albumin was used as a standard with a 50∶1 ratio of 4% (w/v) CuSO_4_.5H_2_O.

### Western Blotting and Immunoprecipitation

All cells were washed twice and scraped into ice cold PBS, where crosslinking were preformed Huh7 cells were cross-linked with dimethyl 3,3′ dithiopropionimidate (DTBP, 5 mM) for 30 min on ice prior to scraping. Cells were pelleted before re-suspending in ice-cold RIPA lysis buffer (0.4% (v/v) with proteinase inhibitor cocktail). Lysates were passed through a 22G needle 5 times and re-cleared by centrifugation at 11600*×g* for 2 min.

For immunoprecipitation, protein-G-Sepharose was pre-cleared by rotation in 5% (w/v) BSA in TBS for 1 h at 4°C, prior to washing 3 times with protein binding buffer (50 mM Tris-HCl pH 7, 50 mM NaCl, 1 µM ZnSO_4_). Samples were incubated with monoclonal anti-β1 integrin antibody (4°C, overnight), pre-cleared protein-G-Sepharose was then added and samples rotated (2 h, 4°C). Bound samples were eluted by heating to 85°C with 1xSDS-PAGE sample buffer. Sepharose beads were pelleted and the eluted supernatant heated to 95°C with β-mercaptoethanol. Where cells had been crosslinked, crosslinking was denatured by heating with DTT before elution (30 min, 37°C).

Proteins were separated by SDS-PAGE and then transferred onto PVDF membranes using 5% (v/v) transfer buffer, 20% (v/v) methanol. The membrane was saturated with blocking solution (TBS 0.1% (v/v) Tween 20, 2% (w/v) BSA, 5% (w/v) dried milk) (1 h, room temp). Membranes were incubated with primary antibody (4°C, overnight). After washing with TBST four times at 10 min intervals the membranes were incubated with secondary antibody for 1 h at room temperature and then washed as before. Bound antibody was detected using the enhanced chemiluminescence system following the manufacturer's instructions. Densitometric analysis was performed using AIDA software.

### Immunostaining

HEK-ACE2 cells were plated onto coverslips and fixed with 4% (w/v) paraformaldehyde (10 min), washed twice with PBS and incubated with blocking buffer (5% BSA in PBS) for 30 min at room temp. Blocking buffer was removed and cells were placed in primary antibodies (ACE2 and ITGB1) for 2 h. Antibody binding was visualised using anti-goat Alexa Fluor 488 and anti-mouse Alexa Fluor 594 (Molecular Probes) for 2 h. Coverslips were mounted using Vectashield (Vector Laboratories Ltd.). Cells were imaged using a Delta Vision microscope and SoftWoRx software.

### Adhesion assay

Adhesion assays were carried out in 96 well plates. Wells were coated overnight (4°C) with protein (10 µg/ml) or PBS and washed in PBS. Wells were then blocked with 1% BSA in serum free media (1 h, 37°C.) Blocking solution was removed and wells washed twice with PBS. Cells were plated at a density of 10,000 or 20,000 cells per well in serum-free medium and allowed to attach by incubation at 37°C for 2.5 h. Non-adherent cells were removed by rinsing wells twice with PBS. Adhered cells were quantified using MTS reagent and measuring absorbance at 492 nm.

### Cell to cell adhesion assay

HEK, HEK-ACE2 or HEK-tACE cells were seeded into a microtitre plate and starved in serum free medium (16 h). Huh7 cells were labelled with BCECF in OptiMEM (37°C, 30 min); labelled cells were washed three times with PBS. Huh7 cells were resuspended in serum free medium and plated onto the HEK, HEK-ACE2 or HEK-tACE cells for 2 h, non-adherent cells were removed by washing in PBS, PBS was added to each well and the fluorescence was read, (excitation 440 nm/emission 535 nm). Cell adhesion was examined using an inverted microscope (TE-2000E, Nikon) illuminated with a halogen lamp filtered through a GFP bandpass filter (450–480 nm excitation wavelength).

### Molecular modelling

Protein structures were taken from the PDB.org, file 1r42 and manipulated with Discovery Studio 2.0 (DS2.0, Accelrys Inc.). Turquoise, ACE2 extracellular domain, residues 1–615; red, tACE; yellow, RGD motif; Mln-4760, green; zinc, lilac; active site residues, pink; all other colours in the ribbon structure are sections of the C-terminal domain disordered in solution. In the spacing filling model: Overall surface, light pink; hydrogen bond acceptors, red; hydrogen bond donors, blue.

### Statistical analysis

Results are expressed as mean +/− standard error of the mean (SEM). Significance was assessed by Student's *t test* or one-way ANOVA and *p≤0.05* was considered significant.
